# Role of Myeloid Tet Methylcytosine Dioxygenase 2 in Pulmonary and Peritoneal Inflammation Induced by Lipopolysaccharide and Peritonitis Induced by *Escherichia coli*

**DOI:** 10.3390/cells11010082

**Published:** 2021-12-28

**Authors:** Wanhai Qin, Xanthe Brands, Hisatake Matsumoto, Joe M. Butler, Cornelis van’t Veer, Alex F. de Vos, Joris J. T. H. Roelofs, Brendon P. Scicluna, Tom van der Poll

**Affiliations:** 1 Center of Experimental & Molecular Medicine, Amsterdam University Medical Centers, Location Academic Medical Center, University of Amsterdam, 1105 AZ Amsterdam, The Netherlands; x.brands@amsterdamumc.nl (X.B.); h.matsumoto0828@gmail.com (H.M.); j.m.butler@amsterdamumc.nl (J.M.B.); c.vantveer@amsterdamumc.nl (C.v.V.); a.f.devos@amsterdamumc.nl (A.F.d.V.); b.scicluna@amsterdamumc.nl (B.P.S.); t.vanderpoll@amsterdamumc.nl (T.v.d.P.); 2 Department of Pathology, Amsterdam University Medical Centers, Location Academic Medical Center, University of Amsterdam, 1105 AZ Amsterdam, The Netherlands; j.j.roelofs@amsterdamumc.nl; 3 Department of Clinical Epidemiology, Biostatistics and Bioinformatics, Amsterdam University Medical Centers, Location Academic Medical Center, University of Amsterdam, 1105 AZ Amsterdam, The Netherlands; 4 Division of Infectious Diseases, Amsterdam University Medical Centers, Location Academic Medical Center, University of Amsterdam, Meibergdreef 9, 1105 AZ Amsterdam, The Netherlands

**Keywords:** TET2, macrophages, inflammation, infection, *E. coli*

## Abstract

Tet methylcytosine dioxygenase 2 (Tet2) mediates demethylation of DNA. We here sought to determine the expression and function of Tet2 in macrophages upon exposure to lipopolysaccharide (LPS), and in the host response to LPS induced lung and peritoneal inflammation, and during *Escherichia (E.) coli* induced peritonitis. LPS induced *Tet2* expression in mouse macrophages and human monocytes in vitro, as well as in human alveolar macrophages after bronchial instillation in vivo. Bone marrow-derived macrophages from myeloid Tet2 deficient (*Tet2^fl/fl^LysM^Cre^*) mice displayed enhanced production of IL-1β, IL-6 and CXCL1 upon stimulation with several Toll-like receptor agonists; similar results were obtained with LPS stimulated alveolar and peritoneal macrophages. Histone deacetylation was involved in the effect of Tet2 on IL-6 production, whilst methylation at the *Il6* promoter was not altered by Tet2 deficiency. *Tet2^fl/fl^LysM^Cre^* mice showed higher IL-6 and TNF levels in bronchoalveolar and peritoneal lavage fluid after intranasal and intraperitoneal LPS administration, respectively, whilst other inflammatory responses were unaltered. *E. coli* induced stronger production of IL-1β and IL-6 by Tet2 deficient peritoneal macrophages but not in peritoneal lavage fluid of *Tet2^fl/fl^LysM^Cre^* mice after in vivo intraperitoneal infection. *Tet2^fl/fl^LysM^Cre^* mice displayed enhanced bacterial growth during *E. coli* peritonitis, which was associated with a reduced capacity of *Tet2^fl/fl^LysM^Cre^* peritoneal macrophages to inhibit the growth of *E. coli* in vitro. Collectively, these data suggest that Tet2 is involved in the regulation of macrophage functions triggered by LPS and during *E. coli* infection.

## 1. Introduction

Innate immune cells mediate the first line of defense against infection. Their response to invading pathogens is fine-tuned in order to effectively eradicate infection and at the same time avoid tissue damage [1]. A dysregulated host response to infection results in organ dysfunction and sepsis, of which the mortality is around 30% [2,3,4]. Thus, investigating the mechanisms that regulate innate immunity is key to understanding host responses during sepsis.

The regulation of innate immune responses can occur at different levels involving both transcriptional and post-transcriptional mechanisms [5,6]. Epigenetics is the study of heritable phenotype alterations that do not involve variations in the DNA sequence. Epigenetic modulation has recently been recognized to play a crucial role during infection and sepsis [7]. DNA methylation, one of the well-studied epigenetic regulatory mechanisms, was altered in blood leukocytes and monocytes from septic patients [8,9]. Moreover, sepsis-associated changes in monocyte DNA methylation occurred in parallel with the acquisition of a tolerized phenotype (reflected by altered responsiveness to endotoxin, a state previously referred to as “endotoxin tolerance”) and were related with organ dysfunction [9]. DNA methylation is established, maintained and regulated by DNA methyltransferases (DNMTs) and Tet methylcytosine dioxygenases (TETs); the expression of these enzymes was altered in blood leukocytes from septic patients [10]. Together, these data suggest that leukocyte DNA methylation and its regulators play important roles during sepsis.

Tet2 was originally recognized for its role in cell differentiation, embryonic development and cancer [11]. *TET2* expression was increased in blood leukocytes during sepsis [10]. Tet2 is involved in the regulation of inflammatory responses in multiple types of immune cells, in both DNA methylation-dependent and independent manners [12,13]. Additionally, Tet2 can repress inflammatory activation of macrophages and promote alternative activation of these cells [14,15]. In mice, in vivo, the inhibitory effect of Tet2 on inflammatory activation of macrophages in atherosclerosis, colitis and peritonitis was regulated through histone deacetylases (HDACs) [16,17]. In contrast, Tet2 was reported to promote neuroinflammatory responses in microglia, tissue macrophages located in the brain [18], suggesting that the role of Tet2 in macrophage activation may depend on the condition and/or tissue environment. Mice with global Tet2 deficiency demonstrated spontaneous bacterial translocation to extra-intestinal organs [19]. However, in abdominal sepsis induced by cecal ligation and puncture, Tet2 deficient mice showed a reduced mortality, possibly through a disrupted emergency myelopoiesis and associated cytokine storm [20]. Of note, these two previous investigations [19,20] made use of mice with a global Tet2 deficiency; thus far mice with a myeloid cell-specific *Tet2* deletion have not been studied in conditions relevant for infection or sepsis.

We sought to determine the role of Tet2 in macrophage activation in response to bacterial stimulation. To this end, we generated myeloid cell-specific Tet2 deficient mice and evaluated innate immune responses of different macrophage subsets upon exposure to bacterial agonists in vitro and of intact mice in vivo after administration of lipopolysaccharide (LPS) or infection with viable *Escherichia (E.) coli*. 

## 2. Methods

### 2.1. Animals

Homozygous *Tet2^fl/fl^* mice [21] were crossed with *LysM^Cre^* mice [22] (Jackson Laboratory, Bar Harbor, ME) to generate myeloid cell-specific Tet2 deficient (*Tet2^fl/fl^LysM^Cre^*) mice. *Tet2^fl/fl^* Cre-negative littermates (*Tet2^fl/fl^* mice) were used as controls in all experiments. *LysM^Cre^* mice have been widely used for the elimination of genes in bone marrow-derived macrophages (BMDMs) and tissue-resident macrophages, including alveolar macrophages (AMs) and peritoneal macrophages (PMs) [23,24]. *Tet2^fl/fl^LysM^Cre^* mice have an unaltered cellular composition of blood and spleen when compared with control mice and unlike global *Tet2*-knockout mice are not prone to develop pre-leukemic myeloproliferation [19]. All genetically modified mice were backcrossed at least eight times to a C57Bl/6 background and age and sex matched when used in experiments. Mice were used at 8–12 weeks of age.

### 2.2. Macrophage Preparation and Stimulation

To obtain BMDMs bone marrow was isolated and cultured with 15% of L929-conditioned medium for seven days for differentiation [25]. BMDMs were then plated in 24-well plates (Greiner bio-one, Alphen a/d Rijn, The Netherlands) overnight before stimulation with 100 ng/mL LPS (from *E. coli* O111:B4; InvivoGen, Toulouse, France), 1 µg/mL Pam3CSK4 (tlrl-pms, InvivoGen), 1 µg/mL lipoteichoic acid (LTA) (tlrl-pslta, InvivoGen), 20 µg/mL polycytidylic acid (poly(I:C)) (tlrl-pic, InvivoGen), 10 μg/mL muramyl dipeptide (MDP) (tlrl-mdp, InvivoGen) or heat-killed *E. coli* (O18:K1; MOI of 10) for defined time periods. Cell supernatant was collected and stored at −20 °C until further analysis; cells were collected for RNA or DNA isolation.

In some experiments, BMDMs were pretreated (2 h) with 100 nM trichostatin A (TSA, R&D Systems, Minneapolis, MN, USA) or 4 μM MS-275 (R&D Systems) before LPS stimulation. The effect of TSA and MS treatment on macrophage viability was measured with flow cytometry after staining with fixable viability dye eFluo 780 (Thermofisher, Waltham, MA, USA). PMs were harvested by peritoneal lavage and seeded in 48-well or six-well flat-bottom culture plates (Greiner bio-one) at a density of approximately 0.5 × 10^6^ cells per well in RPMI complete medium containing 10% FBS, 1% penicillin/streptomycin, 2 mM l-glutamine and 25mM HEPES (Gibco, Thermo Fisher, Waltham, MA, USA) and left to adhere for 3 h. PMs were then washed and stimulated for 12 h with heat-killed *E. coli* (MOI of 10; O18:K1) or 100 ng/mL of ultrapure LPS (*E. coli* O111:B4; InvivoGen). Cell supernatant was collected and stored at −20 °C until further analysis (done within one week), cells were collected for RNA or DNA isolation. In some experiments, PMs were pretreated (2 h) with 100 nM TSA or 4 μM MS-275 before LPS stimulation.

AMs were harvested by bronchoalveolar lavage (BAL) and seeded in 96-wells flat-bottom culture plates (Greiner bio-one) at a density of approximately 5 × 10^4^ cells per well in RPMI complete medium containing 10% FBS, 1% penicillin/streptomycin, 2 mM L-glutamine and 25 mM HEPES (Gibco) and left to adhere for 3 h. AMs were stimulated for 12 h with 100 ng/mL of ultrapure LPS (*E. coli* O111:B4; InvivoGen). Cell supernatant was collected and stored at −20 °C until further analysis (done within one month), cells were collected in RNA isolation buffer provided in the isolation kit and stored in −20 °C for RNA isolation later.

### 2.3. Quantitative Reverse Transcription PCR (qRT-PCR)

qRT-PCR was done according to the MIQE guidelines as previously described [26]. Briefly, total RNA was isolated with NucleoSpin columns (Bioke, Leiden, The Netherlands) according to the manufacturer’s recommendations. cDNA was then prepared using AMV Reverse Transcriptase (Promega, Leiden, The Netherlands) according to manufacturer’s instructions. Gene expression analysis was performed using a Roche LightCycler 480 thermocycler with SensiFAST Real-time PCR kit (#CSA-01190; Bioline, London, UK) using the gene-specific primers listed in Table S1. All results were normalized to *Hprt* expression levels.

### 2.4. Enzyme-Linked Immunosorbent Assay (ELISA)

Murine chemokine (C-X-C motif) ligand (CXCL)1, interleukin (IL)-1β, IL-6, tumor necrosis factor (TNF) and myeloperoxidase (MPO), as well as human CXCL8 were measured by ELISA’s (R&D Systems) according to manufacturer’s description.

### 2.5. Analysis of Publicly Available Datasets

*TET2* expression in human monocytes activated ex vivo with LPS was determined using RNAseq dataset GSE161839, derived from a study from our group [27]. The RNA was isolated from adherent and non-adherent human monocytes before or after 24 h of LPS (10 ng/mL) stimulation (*n* = 6 for each condition) as described in detail [27]. *TET2* expression was determined in human AMs using microarray data deposited in GSE40885, derived from a study from our group [28]. In this study, 6 healthy humans were administered with LPS (4 ng/kg body weight) in one lung segment and normal saline in the contralateral lung via a bronchoscope; 6 h later, a bilateral bronchoalveolar lavage (BAL) was done and RNA was harvested from purified AMs as described in detail [28].

### 2.6. TET2 Overexpression and NF-κB Luciferase Assay

HEK293T cells stably expressing CD14 and Toll-like receptor (TLR)2 (CD14/TLR2-HEK293T cells, generously provided by Dr. D. Golenbock (University of Massachusetts Medical School, Worcester, MA, USA) were plated in 48-well plates (Greiner bio-one) overnight. Full-length TET2 expressing plasmid (provided by Professor Atsushi Kaneda from Chiba University, Chiba, Japan) [29] was transfected using lipofectamine™ 2000 transfection reagent (Invivogen, Toulouse, France) according to manufacturer’s instructions. 24 h after transfection, the cells were stimulated with 1 µg/mL Pam3CSK4 for 12 h, after which supernatants were collected and stored in −20 °C until further analysis.

To monitor the effect of TET2 on nuclear factor-κB (NF-κB) activation triggered by stimulation downstream of TLRs, NF-κB signaling activity was measured by NF-κB luciferase assay. Briefly, HEK293T cells seeded in 96 wells plates (Greiner bio-one) were transfected with 83 ng human myeloid differentiation primary response 88 (MyD88)-HA-tagged (pUNO) expression vector or empty control vector (Invivogen), with the indicated amount of full-length TET2 expressing plasmid or control plasmid, and with 33 ng of NF-kB-Luciferase and 0.7 ng of Renilla-Luciferase constructs in a final volume of 100 µL Opti-Mem. The empty vector control plasmid was added to keep an equal amount of total DNA expression vectors in each well. After 24 h of co-transfection, cell supernatant was collected and stored at −20 °C until further analysis and cells were lysed for NF-κB luciferase assay using the DualGlo kit (Promega, Madison, WI, USA).

### 2.7. Western Blot

Western blot was performed as reported [30]. Briefly, total protein was extracted and separated by 10% SDS gel electrophoresis and transferred to a PVDF membrane (Millipore, Billerica, MA, USA). Membranes were blocked for 1 h in 5% milk in tris-buffered saline (TBS) supplemented with 0.1% Tween 20 (TBST buffer) and incubated overnight with (primary) antibodies against TET2 (ab94580; Abcam, Cambridge, MA, USA), or Beta-Actin (4967L, Cell Signaling Technology, Leiden, The Netherlands) at 4 °C. After incubation with horseradish peroxidase (HRP)-conjugated secondary antibody against rabbit IgG (#7074, Cell Signaling Technology), blots were imaged using Lumilight plus ECL substrate (Roche, Almere, The Netherlands) on an ImageQuant LAS 4000 biomolecular imager (GE Healthcare, Buckinghamshire, UK).

### 2.8. 5-Methylcytosine (5-mC) Dot Blot Assay

DNA methylation levels in BMDMs were detected by dot blot as described before [31]. DNA was isolated from naive BMDMs using DNeasy Blood and Tissue Kit (Qiagen GmbH, Hilden, Germany) and quantified with Nanodrop 2000 (Thermo Scientific, Wilmington, DE, USA). DNA was then denatured, neutralized, serial 2-fold diluted and spotted onto an Hybond-N+ nylon membrane (GE Healthcare, Eindhoven, The Netherlands). After drying, the membrane was exposed to UV light for 30 s at 120,000 μJ/cm^2^ for crosslinking and then blocked in 5% milk TBST for 1 h at room temperature. Thereafter, the membrane was incubated for 1 h at room temperature with monoclonal primary antibodies against m5C (1:1000 dilution, 28692S; Cell Signaling Technology) in 5% milk TBST. After probing with HRP-conjugated secondary antibodies against rabbit IgG (#7074, Cell Signaling Technology), blots were imaged using Lumilight plus ECL substrate (Roche) on an ImageQuant LAS 4000 biomolecular imager (GE Healthcare). The same amount of total DNA (200, 100 and 50 ng) was spotted on another membrane and then was stained with 0.02% methylene blue (Sigma-Aldrich, Amsterdam, The Netherlands) in 0.3 M sodium acetate (pH 5.2).

### 2.9. Global DNA Hydroxymethylation (5-hmC) ELISA Assay

DNA was isolated from naive BMDMs using DNeasy Blood and Tissue Kit (Qiagen). Global 5 hmC levels were measured using the MethylFlash Global DNA Hydroxymethylation (5-hmC) ELISA kit (#P-1032; Epigentek, Farmingdale, NY, USA) according to the manual. The result was reported as the percentage of 5 hmC calculated using the formula generated from an accompanied standard curve.

### 2.10. Methylated DNA Immunoprecipitation (MeDIP) and Hydroxymethylated DNA Immunoprecipitation (hMeDIP)

MeDIP and hMeDIP were performed using Methylamp Methylated DNA Capture Kit (Epigentek, Farmingdale, NY, USA) and EpiQuik Hydroxymethylated DNA Immunoprecipitation (hMeDIP) Kit (Epigentek), respectively, following the manufacturer’s instructions. Briefly, DNA was isolated from PMs using DNeasy Blood & Tissue Kit (Qiagen). Prior to immunoprecipitation, genomic DNA was sonicated with a Diagnode BioRuptor to obtain DNA fragments ranging in size from 200 to 1000 bp, with a mean fragment size of around 300 bp. Methylated DNA and hydroxymethylated DNA were captured from 100 ng and 500 ng of fragmented DNA, respectively. DNA was added to each antibody-coated well and incubated for 120 min at room temperature on an orbital shaker. After treating with proteinase K for 1 h at 65 °C, DNA was eluted from the wells and adjusted to a final volume of 100 µL with nuclease-free water. For each sample, the input sonicated DNA was used for normalization. Input DNA and immunoprecipitated DNA were used to perform qPCR with *Il6* promoter primers (Forward: 5-GTGCTCATGCTTCTTAGGGCT-3′; Reverse 5′-GGGTGGGGCTGATTGGAAAC-3′).

### 2.11. Mouse Models

Lung inflammation was induced by intranasal administration of 1 µg of ultrapure LPS (*E. coli* O111:B4; InvivoGen) in 50 µL saline as described [32]. Mice were euthanized 6 h post-inoculation by heart puncture after injection of ketamine/medotomidine as described [32]. Briefly, the right lung was used for BAL by instilling 2 × 0.5 mL of sterile phosphate-buffered saline; the left lung was preserved for histopathology after fixation in 10% formalin. Cell counts in BAL Fluid (BALF) were determined using a hemocytometer (Beckman Coulter, Fullerton, CA, USA); different cell populations in BALF samples were determined by flow cytometry (details see below) [33]. BALF supernatants were stored at −20 °C until further analysis.

Peritonitis was induced by intraperitoneal injection of 2 mg/kg ultrapure LPS (*E. coli* O111:B4; InvivoGen). Six hours after inoculation, the mice were euthanized as described above. Peritoneal lavage was performed by instilling 5 mL of sterile phosphate-buffered saline. Cell counts in peritoneal lavage fluid (PLF) were determined using a hemocytometer (Beckman Coulter); different cell populations in PLF samples were determined by flow cytometry (details see below). The PLF and organ homogenate supernatants were stored at −20 °C until further analysis.

Abdominal infection was induced by intraperitoneal injection of approximately 1 × 10^4^ CFU *E. coli* (O18:K1) as described [34,35]. Fifteen minutes before and 12 h after infection (if applicable), mice were administered with buprenorphine subcutaneously (0.05 mg/kg) for pain relief. Mice were euthanized 6 or 16 h after infection; PLF was harvested and processed in the same way as described in LPS peritonitis model. Organs were harvested and homogenized for determining cytokine production and bacterial burdens as described [34,35]. Bacterial loads in PLF and organs were determined by serial dilutions and plating on blood agar plates.

### 2.12. Flow Cytometry

Flow cytometry was done on FACS Calibur (Becton Dickinson, Franklin Lakes, NJ, USA) and data were analyzed using FlowJo software (Becton Dickinson) as described [33]. BALF and PLF cells were resuspended in FACS buffer (0.5% BSA, 0.35 mM EDTA, 0.01% NaN3). Staining of cells was performed according to the manufacturer’s recommendations using fixable viability dye eFluor 780, rat anti-mouse-CD16/CD32 (clone 93), rat anti-mouse-CD45 PE-eFluor610 (30-F11), rat anti-mouse CD11b PE-Cy7 (clone M1/70), rat anti-mouse Siglec-F Alexa Fluor 647 (clone E50-2440; BALF cells only), rat anti-mouse Ly-6C Alexa Fluor 700 (clone AL-21) (all from BD Biosciences), rat anti-mouse Ly-6G FITC (clone 1A8; Biolegend, San Diego, CA, USA) and rat anti-mouse F4/80 APC (BM8; PLF cells only). Examples of the gating strategy for BALF leukocytes and PLF leukocytes are depicted in Figure S1.

### 2.13. Histology

The paraffin-embedded left lung lobe was cut into four-micrometer sections and stained with hematoxylin and eosin (H&E). Slides were coded and scored by a pathologist blinded for group identity with respect to the following parameters: bronchitis, edema, interstitial inflammation, intra-alveolar inflammation, pleuritis, endothelialitis, and percentage of the lung surface demonstrating confluent inflammatory infiltrate as described [36]. Each parameter was graded 0–4, with 0 being ‘absent’ and 4 being ‘severe’; the total pathology score was expressed as the sum of the score for all parameters.

### 2.14. Clinical Chemistry Assays

Aspartate aminotransferase (AST), alanine aminotransferase (ALT) and lactate dehydrogenase (LDH) were measured using a c702 Roche Diagnostics analyzer (Roche Diagnostics, Almere, The Netherlands).

### 2.15. In Vitro E. coli Growth Inhibition Assay

PMs cells were seeded in 96-well flat-bottom culture plates (Greiner bio-one) at a density of approximately 0.2 × 10^6^ cells per well in antibiotics-free RPMI complete medium containing 10% murine serum, 2 mM L-glutamine and 25mM HEPES (Gibco). Approximately 1000 *E. coli* (O18:K1) opsonized with 30% normal mouse serum prepared from wild type mice were added to PMs and centrifuged for 5 min at 4 °C with 500*× g* to improve bacteria-cell contact. After incubation for 0, 1 or 3 h in a cell culture incubator, cell culture supernatants were collected and PMs were lysed with sterile water at room temperature for 20 min. Cell lysates and supernatants were pooled tenfold and serially diluted before plating on blood agar plates. Colony counts were enumerated by plating serial dilutions on blood agar plates.

### 2.16. Statistical Snalysis

Statistical analyses were performed by a paired *t*-test or non-parametric *t*-test (Mann–Whitney U -est) where appropriate. Analysis was done using GraphPad Prism version 8 (GraphPad Software, San Diego, CA, USA). Statistical significance is shown as * *p* < 0.05; ** *p* < 0.01; *** *p* < 0.001; **** *p* < 0.0001.

## 3. Results

### 3.1. LPS Induces Expression of Tet2/TET2 mRNA in Mouse and Human Macrophages

**Figure 1:**
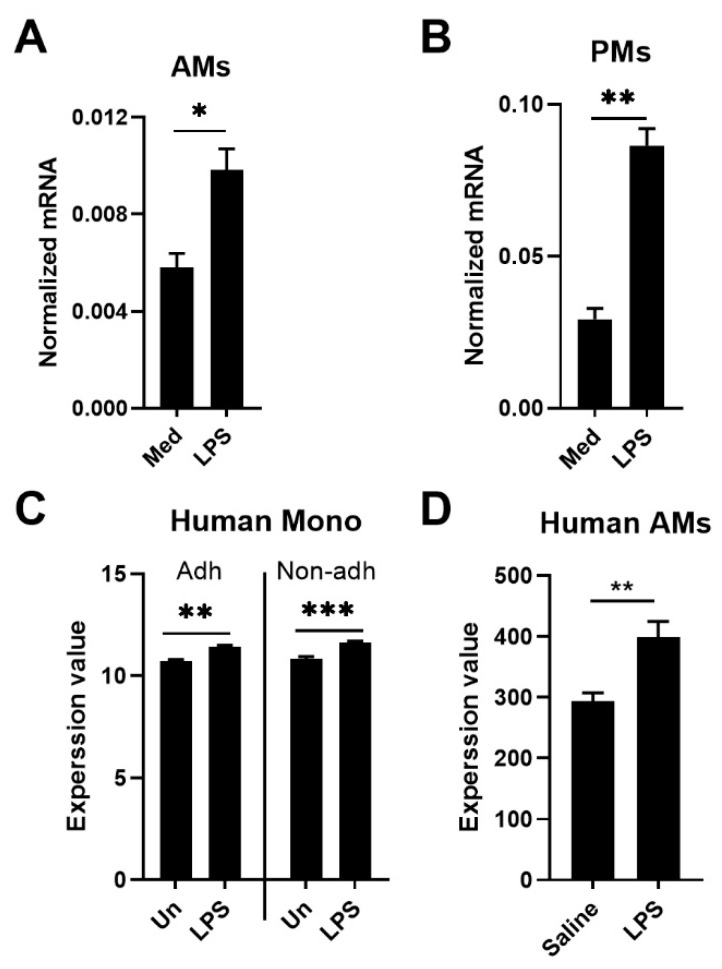
LPS induces expression of *Tet2/TET2* in mouse and human macrophages. (**A**) *Tet2* expression in murine alveolar macrophages (AMs) after stimulation with 100 ng/mL LPS for 12 h; (**B**) *Tet2* expression in murine peritoneal macrophages (PMs) after stimulation with 100 ng/mL LPS for 12 h; (**C**) *TET2* expression in adherent (Adh) and non-adherent (Non-adh) human monocytes (Human mono) before (Un) and 24 h after stimulation with 10 ng/mL LPS; (**D**) *TET2* expression in human AMs collected 6 h after bronchial instillation of LPS (4 ng/kg body weight) or (in the contralateral lung) saline. mRNA expression was normalized to HPRT mRNA (**A**,**B**). *n* = 6. Data is shown as bar graphs with mean ± SEM. The *p* values were calculated using Mann–Whitney test (**A**,**B**,**D**) or paired *t*-test (**C**). * *p* < 0.05, ** *p* < 0.01, *** *p* < 0.001.

To obtain insight in changes in Tet2 mRNA levels during macrophage activation, we first stimulated mouse AMs and PMs with LPS in vitro and measured *Tet2* expression (Figure 1A,B). Macrophages from both body sites showed increased Tet2 mRNA levels upon LPS exposure. To determine whether *TET2* expression is regulated similarly in human cells, we made use of publicly available datasets derived from previous studies from our group [27,28]. Primary human monocytes, either adherent or non-adherent, stimulated with LPS demonstrated increased TET2 mRNA levels relative to monocytes cultured in medium only (Figure 1C; GSE161839) [27]. Likewise, human AMs harvested after an in vivo bronchial LPS challenge in healthy subjects showed increased TET2 mRNA levels when compared with AMs obtained from the contralateral control lung administered with normal saline (Figure 1D, GSE40885) [28]. Taken together, these data show that *Tet2/TET2* is upregulated in mouse and human macrophages, as well as human monocytes, after exposure to LPS in vitro or in vivo.

### 3.2. Tet2 Inhibits Il1b, Il6 and Cxcl1 Expression by Macrophages Stimulated with Various TLR/NOD2 Ligands

**Figure 2:**
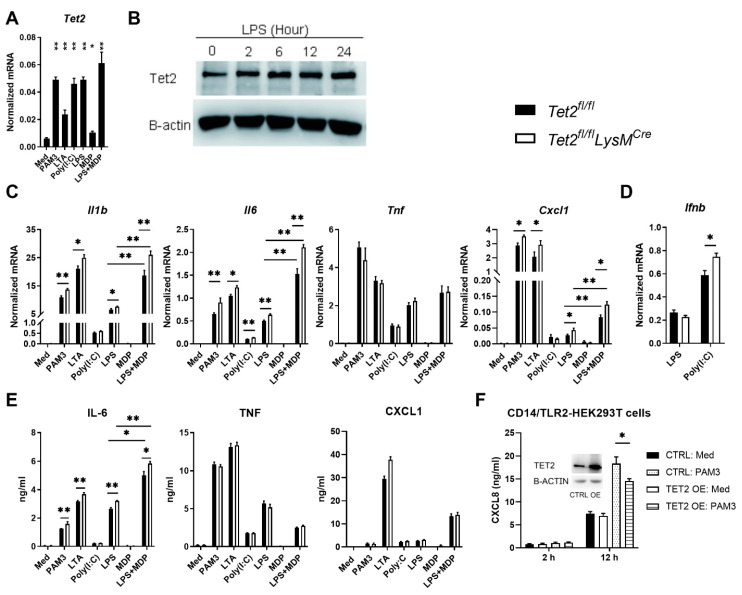
Tet2 represses *Il1b*, *Il6* and *Cxcl1* expression in macrophages stimulated with TLR/NOD2 ligands. (**A**) *Tet2* expression in murine bone marrow-derived macrophages (BMDMs) after stimulation with medium control, PAM3, LTA, Poly(I:C), MDP or MDP plus LPS for 12 h; (**B**) BMDMs were stimulated with LPS for different time points, Tet2 protein in BMDMs were then detected by western blot, the relative Tet2 protein levels were estimated by normalizing to beta-actin (Ration) using ImageJ based on the densitometry; (**C**) *Ilb*, *Il6*, *Tnf* and *Cxcl1* expression in BMDMs from *Tet2^fl/fl^LysM^Cre^* (open bars) and control mice (black bars) after stimulation with medium control, LPS, PAM3,LTA, Poly(I:C), MDP or MDP plus LPS for 12 h; (**D**) *Ifnb* expression in BMDMs from *Tet2^fl/fl^LysM^Cre^* and control mice after stimulation with LPS or Poly(I:C) for 12 h; (**E**) IL-6, TNF and CXCL1 levels in supernatants of BMDMs from *Tet2^fl/fl^LysM^Cre^* and control mice after stimulation with medium control, LPS, PAM3,LTA, Poly(I:C), MDP or MDP plus LPS for 12 h; (**F**) CXCL8 production by CD14/TLR2-HEK293T cells after stimulation with PAM3CSK4 (1 µg/mL) for 2 or 12 h; TET2 OE indicates CD14/TLR2-HEK293T cells transfected with TET2 expressing plasmid 24 h before stimulation, CTRL indicates CD14/TLR2-HEK293T cells transfected with control plasmid 24 h before stimulation; the insert figure shows TET2 protein levels in CD14/TLR2-HEK293T cells 24 h after transfection with human TET2 expressing plasmid (OE) or control plasmid (CTRL). mRNA expression was normalized to HPRT mRNA (**A**–**C**). *n* = 6. Data is shown as bar graphs with mean ± SEM. *p* values were calculated using Mann–Whitney test. * *p* < 0.05, ** *p* < 0.01.

To determine whether *Tet2* also can be induced by agonists of other common pattern recognition receptors (PRRs) besides LPS (which stimulates TLR4), murine BMDMs were incubated with PAM3CSK4 (TLR1/2), LTA (TLR2), Poly(I:C) (TLR3) or MDP (NOD2) (Figure 2A). All stimuli increased *Tet2* expression, which is consistent with previous studies reporting that *Tet2* can be induced by activation of NF-κB and MyD88 dependent signaling [14,15]. Akin to primary AMs and PMs, LPS induced *Tet2* in BMDMs, which was further enhanced by the addition of MDP. Furthermore, an increase of Tet2 protein levels in BMDMs was detected from 2 h after LPS stimulation and maintained until 24 h (Figure 2B). To investigate the role of Tet2 in macrophage activation, we incubated BMDMs generated from bone marrow of myeloid cell-specific *Tet2* knockout (*Tet2^fl/fl^LysM^Cre^*) mice and littermate control (*Tet2^fl/fl^*) mice with various PRR ligands and measured the expression of *Il1b*, *Il6, Cxcl1* and *Tnf*. LTA and LPS induced higher expression of *Il1b*, *Il6* and *Cxcl1* in *Tet2* knockout macrophages (Figure 2C). While MDP did not increase cytokine mRNA’s to a significant extent, the addition of MDP to LPS resulted in synergistic induction of *Il1b*, *Il6* and *Cxcl1*, corroborating previous studies [37,38,39], which was further enhanced in *Tet2* knockout macrophages (Figure 2C). TLR3 signals via a different (non-MyD88 dependent) route when compared with other TLRs, resulting in amongst other induction of type I interferons [40]. BMDM Tet2 deficiency was also associated with enhanced *Il6* and *Ifnb* expression upon stimulation with the TLR3 agonist Poly(I:C) (Figure 2C,D). Expression of *Tnf* was not influenced by Tet2 deficiency irrespective of the stimulus, which is consistent with a previous study [16]. Tet2 deficient BMDMs released more IL-6 protein when compared with control BMDMs, whilst CXCL1 and TNF release was similar between genotypes (Figure 2E). BMDMs did not release IL-1β upon LPS stimulation. To further investigate the effect of Tet2 on TLR-induced cell activation we overexpressed human TET2 in CD14/TLR2-HEK293T cells. TET2 overexpression (confirmed by western blot as shown in the inset of Figure 2E) significantly reduced CXCL8 production induced by PAM3CSK4 (Figure 2F). Together, these data suggest that TET2 represses the transcription of certain cytokine/chemokine genes in response to TLR stimulation.

### 3.3. HDACs Are Involved in Tet2 Mediated Regulation of IL-6 Production

**Figure 3:**
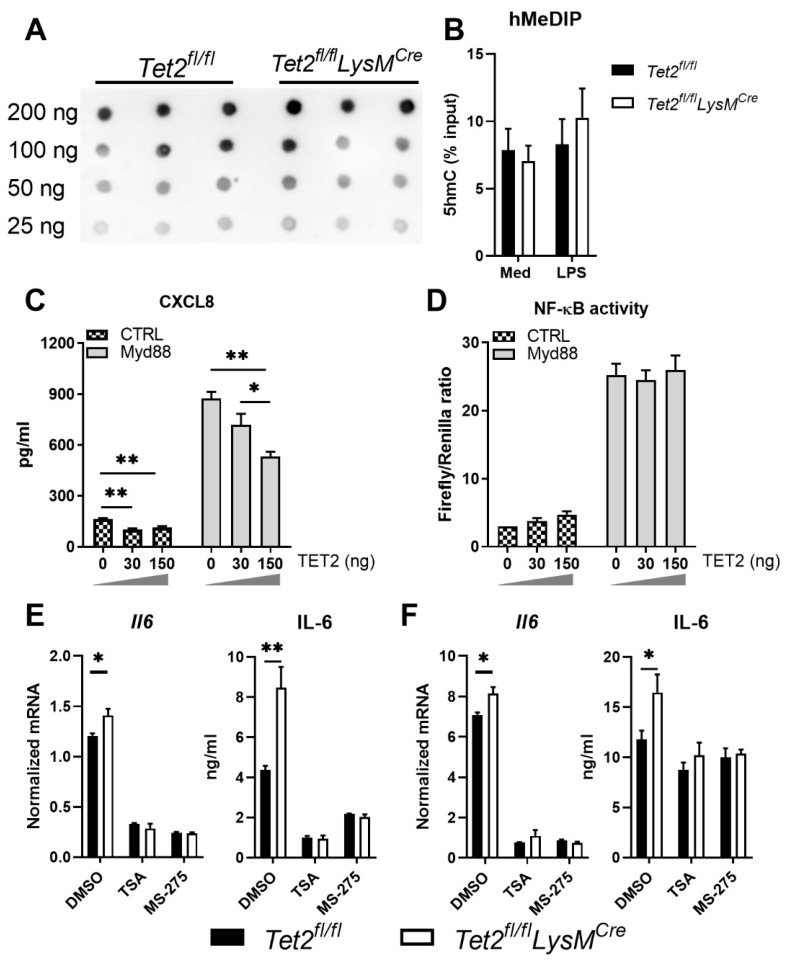
HDACs are involved in TET2-mediated regulation of IL-6 production. (**A**) DNA methylation levels in naive bone marrow-derived macrophages (BMDMs) from *Tet2^fl/fl^LysM^Cre^* and control mice measured by dot blots; (**B**) 5-hydroxymethylcytosine (5hmC) levels measured by hydroxymethylated DNA immunoprecipitation (5hMeDIP) at the *Il6* promoter in peritoneal macrophages (PMs) from *Tet2^fl/fl^LysM^Cre^* (open bars) and control mice (black bars) stimulated with LPS or medium control for 12 h; (**C**,**D**) CXCL8 production by and NF-κB activity in HEK293T cells co-transfected with MyD88 and increasing amounts of TET2 expression vector (0, 30 or 150 ng) 24 h earlier; (**E**,**F**) IL-6 protein production by BMDMs (**E**) and PMs (**F**) from *Tet2^fl/fl^LysM^Cre^* and control mice after stimulation with LPS (100 ng/mL) for 12 h following the pretreatment of 100 nM TSA or 4 μM MS-275. *n* = 3 for (**A**–**D**), *n* = 6 for (**E**,**F**). Data is shown as bar graphs with mean ± SEM. *p*-values were calculated using Mann–Whitney test. * *p* < 0.05, ** *p* < 0.01.

Tet2 is one of the major enzymes for de novo DNA demethylation and has been reported to regulate cytokine expression via DNA methylation [12,13,41]. Therefore, to gain insight into the underlying mechanism by which Tet2 influences TLR responsiveness we first analyzed global DNA methylation levels of unstimulated Tet2 deficient and control BMDMs by dot blot assay, the same amount of the loading DNA was validated by methylene blue staining (Figure S2A). However, Tet2 deficiency did not affect DNA methylation in BMDMs (Figure 3A) to a significant extent. Likewise, 5 hmC levels in BMDMs were not affected by Tet2 deficiency (Figure S2B). Furthermore, the 5-hmC levels at the promoter of *Il6* measured by 5hMeDIP was not affected by Tet2 deficiency in PMs (Figure 3B), although *Il6* expression was significantly increased by Tet2 deficiency in these cells (see below). Additionally, the expression of TET family methylcytosine dioxygenases *Tet1* and *Tet3* was comparable in Tet2-deficient and control BMDMs (Figure S2C). Therefore, these data suggest that Tet2 regulates *Il6* expression independent of modifications in DNA methylation. Since the expression of most inflammatory genes is triggered by NF-κB signaling, we next wondered whether Tet2 deficiency affects NF-κB activity. To test this, we simultaneously overexpressed human TET2 (Figure S3) and MyD88, the major downstream signaling molecule for all TLRs except TLR3, in NF-κB reporter (luc)-HEK293T cells. Overexpression of MyD88 resulted in increased NF-κB activity and CXCL8 release (Figure 3C,D). TET2 overexpression inhibited MyD88 induced CXCL8 production in a dose-dependent manner (Figure 3C), in agreement with the reduced PAM3CSK4-induced CXCL8 production in TET2 transfected CD14/TLR2-HEK293T cells (Figure 2E). Of note, TET2 overexpression did not alter MyD88 induced NF-κB activity (Figure 3D). Previous studies reported that TET2 recruits HDAC1 and HDAC2 to the *Il6* promoter and represses its transcription via histone deacetylation [16,42]. To evaluate a possible role of histone deacetylation in the effect of Tet2 on IL-6 production, Tet2 deficient and control BMDMs and PMs were treated with the HDACs inhibitors TSA or MS-275 (or vehicle) prior LPS stimulation (Figure 3E,F). Both TSA and MS-275 decreased LPS-induced *Il6* expression and IL-6 production by Tet2 deficient and control BMDMs and PMs, and abrogated the difference in IL-6 production between Tet2 deficient and control cells without affecting cell viability (Figure S4). Taken together, these results suggest that histone deacetylation may be involved in Tet2 mediated inhibition of IL-6 production by macrophages.

### 3.4. Tet2 Deficiency in AMs Enhances LPS Induced Cytokine Gene Expression In Vitro but Only Modestly Influences LPS-Induced Lung Inflammation In Vivo

**Figure 4:**
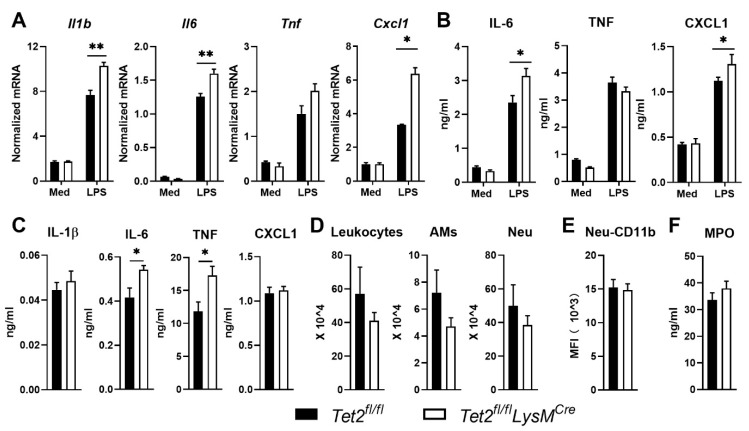
Impact of myeloid Tet2 deficiency on LPS-induced responses by alveolar macrophages in vitro and intact lungs in vivo. (**A**) *Ilb*, *Il6, Tnf* and *Cxcl1* expression in alveolar macrophages (AMs) from *Tet2^fl/fl^LysM^Cre^* (open bars) and control mice (black bars) after stimulation with medium control or LPS for 12 h; mRNA expression was normalized to Hprt mRNA; (**B**) IL-6, TNF and CXCL1, production by AMs from *Tet2^fl/fl^LysM^Cre^* and control mice after stimulation with medium control or LPS for 12 h; (**C**) IL-1β, IL-6, TNF and CXCL1 protein levels, (**D**) number of total leukocytes, AMs and neutrophils (Neu), (**E**) neutrophil CD11b expression and (**F**) MPO levels in bronchoalveolar lavage fluid from *Tet2^fl/fl^LysM^Cre^* and control mice 6 h after administration of LPS (1 µg/mouse) via the airways. *n* = 6 for (**A**,**B**), *n* = 8 for (**C**–**F**). Data are shown as bar graphs with mean ± SEM. *p*-values were calculated using Mann–Whitney test. * *p* < 0.05, ** *p* < 0.01.

Having shown increased *Tet2* expression in LPS-stimulated mouse AMs (Figure 1A) and increased *TET2* expression in human AMs after exposure to LPS in vivo (Figure 1D), we next determined the role of Tet2 in cytokine production by AMs. To this end AMs were isolated from *Tet2^fl/fl^LysM^Cre^* and *Tet2^fl/fl^* control mice and stimulated ex vivo with LPS. Akin to results obtained with BMDMs (Figure 2), Tet2 deficiency in AMs promoted LPS-induced *Il1b, Il6* and *Cxcl1* but not *Tnf* expression (Figure 4A). In agreement, Tet2 deficient AMs released more IL-6 and CXCL1 compared to control AMs, while the TNF release was not different between genotypes (Figure 4B). AMs did not release IL-1β upon LPS stimulation; for this, an additional inflammasome-activating agent would have been needed [43], which was not added. AMs play a crucial role in maintaining homeostasis and regulating inflammation in the lung [44]. Thus, we next evaluated the function of myeloid Tet2 in lung inflammation elicited by LPS administration via the airways in mice in vivo. *Tet2^fl/fl^LysM^Cre^* mice had higher IL-6 and TNF levels in BALF 6 h after local LPS instillation when compared with *Tet2^fl/fl^* control mice, whereas IL-1β and CXCL1 levels were not different between groups (Figure 4C). Total leukocyte and AM numbers did not differ between mouse strains (Figure 4D). Neutrophil influx into BALF and neutrophil activation (measured by CD11b expression and MPO release) were also similar in LPS challenged *Tet2^fl/fl^LysM^Cre^* and control mice (Figure 4D,E). LPS induced modest histopathological changes, which did not differ between *Tet2^fl/fl^LysM^Cre^* and *Tet2^fl/fl^* control mice (Supplementary Figure S5). Together these data suggest that while Tet2 affects cytokine gene expression in AMs, myeloid cell Tet2 deficiency only modestly influences inflammatory responses induced by LPS in mouse lungs in vivo.

### 3.5. Myeloid Tet2 Deficiency Enhances LPS-Induced Cytokine Release by Peritoneal Macrophages In Vitro and during LPS Induced Peritonitis In Vivo

**Figure 5:**
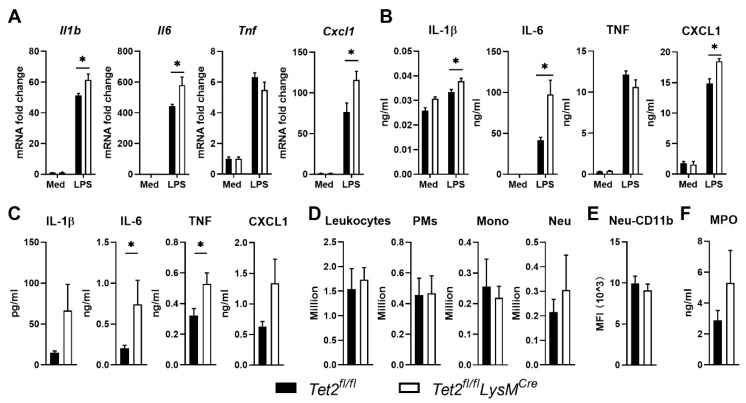
Myeloid Tet2 deficiency enhances IL-6 and TNF release during LPS induced peritonitis. (**A**) *Ilb*, *Il6, Tnf* and *Cxcl1* expression in peritoneal macrophages (PMs) from *Tet2^fl/fl^LysM^Cre^* (open bars) and control mice (black bars) after stimulation with medium control or LPS for 12 h; mRNA expression was normalized to Hprt mRNA; (**B**) IL-1β, IL-6, TNF and CXCL1 production by PMs from *Tet2^fl/fl^LysM^Cre^* and control mice after stimulation with medium control or LPS for 12 h; (**C**) IL-1β, IL-6, TNF and CXCL1 protein levels, (**D**) number of total leukocytes, PMs, monocytes (Mono) and neutrophils (Neu), (**E**) neutrophil CD11b expression and (**F**) MPO levels in peritoneal lavage fluid of from *Tet2^fl/fl^LysM^Cre^* and control mice 6 h after intraperitoneal administration of LPS (2 mg/kg). *n* = 6 for (**A**,**B**), *n* = 8 for (**C**–**F**). Data is shown as bar graphs with mean ± SEM. *p* values were calculated using Mann–Whitney test. * *p* < 0.05.

PMs represent the main resident leukocyte subpopulation in the peritoneal cavity at homeostasis [45]. To determine the role of Tet2 in PMs, PMs were harvested from *Tet2^fl/fl^LysM^Cre^* and *Tet2^fl/fl^* control mice and stimulated with LPS in vitro. Tet2-deficient PMs demonstrated enhanced *Il1b, Il6* and *Cxcl1* expression, and released more IL-1β, IL-6 and CXCL1, upon LPS stimulation, while TNF induction was not affected by Tet2 deficiency at either transcriptional or translational level (Figure 5A,B). To gain insight in the role of macrophage Tet2 in LPS-induced peritoneal inflammation in vivo, *Tet2^fl/fl^LysM^Cre^* and control mice were injected with LPS intraperitoneally; 6 h thereafter cytokine levels and leukocyte numbers were determined in PLF. *Tet2^fl/fl^LysM^Cre^* mice had higher IL-6 and TNF concentrations in PLF when compared with control mice, whilst IL-1β (*p* = 0.16) and CXCL1 (*p* = 0.08) levels tended to be higher (Figure 5C). Leukocyte cell numbers and neutrophil activation (measured as CD11b expression and MPO levels) in PLF were not different between mouse strains (Figure 5D–F). Collectively, these data suggest that Tet2 represses cytokine gene expression in PMs exposed to LPS as well as peritoneal cytokine release during LPS-induced peritonitis without affecting cell influx into the peritoneal cavity.

### 3.6. Myeloid Tet2 Deficiency Reduces Antibacterial Defense during E. coli Induced Peritonitis

**Figure 6:**
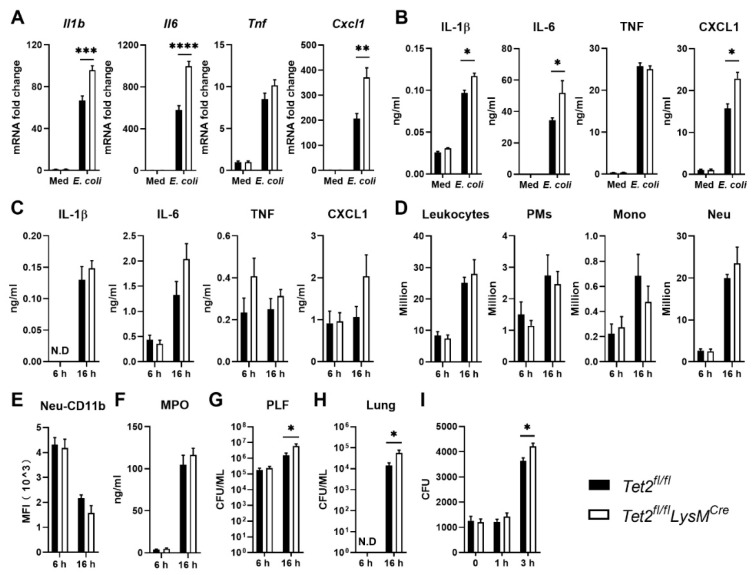
Myeloid Tet2 deficiency impairs antibacterial defense during *E. coli* induced peritonitis. (**A**) *Ilb*, *Il6, Tnf* and *Cxcl1* expression in peritoneal macrophages (PMs) from *Tet2^fl/fl^LysM^Cre^* (open bars) and control mice (black bars) after stimulation with medium control or heat killed *E. coli* (MOI = 10) for 12 h; mRNA expression was normalized to Hprt mRNA; (**B**) IL-1β, IL-6, TNF and CXCL1 production by PMs from *Tet2^fl/fl^LysM^Cre^* and control mice after stimulation with medium control or heat killed *E. coli* (MOI = 10) for 12 h; (**C**) IL-1β, IL-6, TNF and CXCL1 protein levels, (**D**) number of total leukocytes, PMs, monocytes (Mono) and neutrophils (Neu), (**E**) neutrophil CD11b expression and (**F**) MPO levels in peritoneal lavage fluid (PLF) from *Tet2^fl/fl^LysM^Cre^* and control mice intraperitoneally infected with 10^4^ *E. coli* 6 or 16 h earlier; (**G**,**H**) Bacterial burdens in PLF and lung tissue from *Tet2^fl/fl^LysM^Cre^* and control mice intraperitoneally infected with 10^4^ *E. coli* 6 or 16 h earlier; (**I**) Bacterial numbers after coculturing of *E. coli* and PMs from *Tet2^fl/fl^LysM^Cre^* and control mice in vitro up to 3 h. *n* = 6 for (**A**,**B**,**I**), *n* = 8 for (**C**–**F**). Data is shown as bar graphs with mean ± SEM. *p*-values were calculated using Mann–Whitney test. * *p* < 0.05, ** *p* < 0.01, *** *p* < 0.001, **** *p* < 0.0001.

LPS is a common virulence factor of gram-negative bacteria, among which *E. coli* is one of the major pathogens involved in peritonitis [35], and peritonitis is the second most common cause of sepsis [46,47,48]. Akin to results obtained after LPS stimulation (Figure 5A,B), Tet2-deficient PMs exposed to *E. coli* showed increased *Il1b, Il6* and *Cxcl1* expression, and IL-1β, IL-6 and CXCL1 protein release, when compared with control PMs, while induction of TNF was not affected (Figure 6A,B). To obtain insight into the role of macrophage Tet2 in bacterial peritonitis in vivo, *Tet2^fl/fl^LysM^Cre^* and control mice were infected with viable *E. coli* through intraperitoneal injection and euthanized at 6 or 16 h thereafter to study inflammatory responses and bacterial loads. Cytokine levels (Figure 6C), leukocyte counts and neutrophil activation (Figure 6D–F) in PLF were not different between mouse strains at either time point. While at 6 h post-infection, bacterial burdens were not different between mouse strains in any body site, *Tet2^fl/fl^LysM^Cre^* mice had higher bacterial loads in PLF and lungs at 16 h post-infection when compared with control mice (Figure 6G,H). The *E. coli* strain used in this study is resistant to phagocytosis and killing by phagocytes [49]; thus, we next evaluated the bacterial growth-inhibiting capacity of PMs from *Tet2^fl/fl^LysM^Cre^* and *Tet2^fl/fl^* control mice in cocultures with viable *E. coli* in vitro (Figure 6I). *E. coli* numbers increased in the presence of PMs from both mouse strains beyond 1 h of incubation, but to a larger extent in cultures with Tet2 deficient PMs, suggesting that Tet2 contributes to macrophage-mediated bacterial growth inhibition. To explore possible underlying mechanisms, we tested the expression of genes related to antimicrobial effector functions in Tet2 deficient and control PMs after *E. coli* stimulation, i.e., *Rnase6* (encoding ribonuclease A family member K6), *Lcn2* (lipocalin-2), *Hamp* (hepcidin antimicrobial peptide), *Camp* (cathelicidin antimicrobial peptide) and *Cat* (catalase). The expression of none of those genes was changed by Tet2 deficiency, although they were induced by *E. coli* (Figure S6A). In addition, we also evaluated the expression of genes related to ROS or NOS production, i.e., *Nos2* (nitric oxide synthase 2), *Nox2* (NADPH oxidase 2), *Sod1* (superoxide dismutase), *Gpx4* (glutathione peroxidase 4) and *Ptx3* (pentraxin 3). Of these, the expression of *Nos2, Sod1* and *Ptx3* was increased by *E. coli*, but not significantly altered by Tet2 deficiency, although the expression of *Nos2* and *Sod1* tended to be lower in Tet2 deficient peritoneal macrophages (Figure S6B). This model of *E. coli* peritonitis is associated with (late) distant organ injury and sepsis [35]. To evaluate a possible role of myeloid cell Tet2 herein we measured the plasma of AST, ALT (both reflecting hepatocellular injury) and LDH (reflecting cell injury in general) (Supplementary Figure S7A–C), and evaluated lung injury by assessing lung histopathology (Supplementary Figure S7D,E) 16 h after infection. None of these parameters differed between *Tet2^fl/fl^LysM^Cre^* and control mice. Collectively, these data suggest that Tet2 is involved in antibacterial defense mediated by PMs during *E. coli* induced peritonitis.

## 4. Discussion

**Figure 7:**
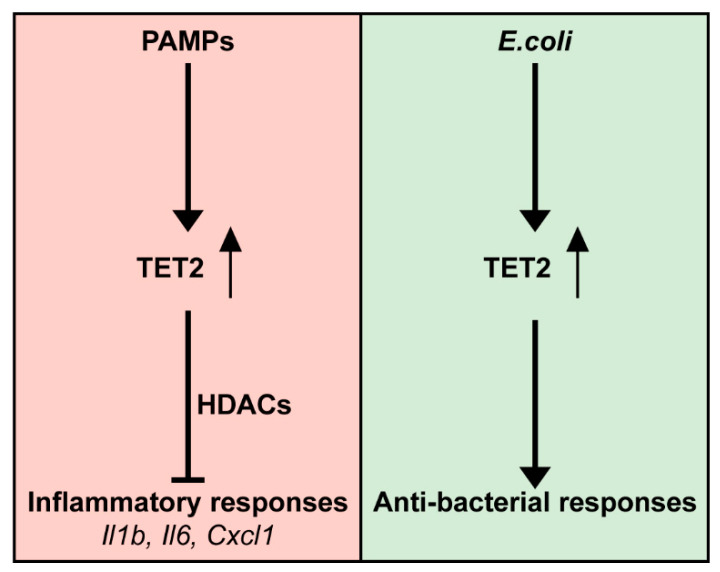
The role of TET2 in myeloid cells during PAMPs stimulation and *E. coli* infection. PAMPs stimulation induces TET2 expression, resulting in downregulation of inflammatory mediators (such as *Il1b*, *Il6* and *Cxcl1*) in a HDACs dependent manner; TET2 is induced during *E. coli* infection and contributes to myeloid cell-mediated host anti-bacterial defense. PAMPs: Pathogen-associated molecular pattern molecules; TET2: Tet methylcytosine dioxygenase 2; HDACs: Histone deacetylases.

Tet2 is an enzyme that mediates the demethylation of DNA and has been widely recognized to play a crucial role in hematopoiesis, particularly myelopoiesis [50]. More recent evidence has implicated Tet2 as a mediator in immune cell activation [51]. We here show that *Tet2*/*TET2* is induced in primary mouse and human macrophages upon stimulation with LPS and that myeloid cell-specific Tet2 deficiency promotes the production of proinflammatory cytokines, most notably IL-6, triggered by LPS in vitro and in the bronchoalveolar and peritoneal compartments of mice in vivo. Moreover, we demonstrate a role for macrophage Tet2 in limiting the growth of *E. coli* in vitro, which was associated with increased growth and dissemination of *E. coli* in mice with myeloid cell-specific Tet2 deficiency in a model of peritonitis and abdominal sepsis (Figure 7).

*Tet2* has been reported to be induced by NF–κB activation (14). In agreement, we show that multiple TLR ligands induced the expression of *Tet2* in macrophages. Of interest, in vivo administration of LPS into the human lung was associated with increased *TET2* expression in AMs, suggesting that our findings in myeloid cell Tet2 deficient mice might have relevance for inflammatory lung diseases in humans. In support of a role of TET2 in the regulation of inflammatory reactions in human cells, *TET2* mutations in human macrophages prepared from bone marrow of patients with myelodysplastic syndromes were associated with increased induction of *IL6* upon LPS stimulation (14). Likewise, TET*2*-silenced human dendritic cells showed enhanced LPS-induced *IL6* expression [16]. Together these data indicate that the phenotype of Tet2 deficient mouse macrophages may be informative for the role of TET2 in human cells. Noteworthy, unlike the frequently reported somatic TET2 mutation, heterozygous germline TET2 mutations did not alter cytokine or chemokine secretion by human monocytes [52,53]. Previous investigations documented that Tet2 deficient murine BMDMs and bone marrow-derived dendritic cells display enhanced *Il6* induction upon stimulation with LPS [16]. Moreover, Tet2 deficiency was reported to contribute to IL-1β production in stimulated macrophages by enhancing NLRP3 inflammasome priming in addition to promoting *Il1b* gene transcription [17]. We here report that Tet2 deficient BMDMs have enhanced *Il6*, *Il1b* and *Cxcl1* expression upon stimulation with multiple TLR agonists (TLR1/2, TLR2, TLR3, TLR4). Noteworthy, *IFNb* expression induced by the TLR3 ligand poly(I:C), which unlike all other TLRs does not signal via MyD88, was also increased in Tet2 deficient BMDMs. Considering the important role of type I interferons in the host response to viral infection [54], these data suggest Tet2 might play a role herein. Additionally, we show that primary macrophages from the bronchoalveolar space (AMs) and peritoneal cavity (PMs) from *Tet2^fl/fl^LysM^Cre^* mice display increased LPS-induced *Il6*, *Il1b* and *Cxcl1* expression. In a reversed approach, we demonstrate that overexpression of TET2 inhibits CXCL8 release by HEK293T/TLR2 cells stimulated with PAM3CSK4, as well as by HEK293T cells with forced expression of MyD88. Jointly, these data indicate that Tet2 acts as an inhibitory regulator of gene expression of several cytokines and chemokines in cells exposed to stimuli with relevance for infection.

As Tet2 was originally reported to be an “eraser” of DNA methylation modification that removes methyl groups via stepwise oxidization, the best-known mechanism by which Tet2 regulates gene expression is through DNA demethylation [11]. Moreover, Tet2 was reported to regulate cytokine production in some immune cells by modifying DNA methylation, such as T cells [12] and dendritic cells [13]. We here provide evidence that Tet2 deficiency does not affect 5-hmC levels at the *Il6* promoter in macrophages, arguing against a role for Tet2 mediated demethylation of DNA, which is in agreement with previous findings that DNA methylation was not affected by Tet2 deficiency in LPS activated microglia, resident macrophages in brain [18]. Tet2 overexpression did not influence NF-κB activity in HEK293T cells transfected with MyD88, whilst it did inhibit CXCL8 production, suggesting Tet2 functions directly at its target genes. Indeed, Tet2 mediated transcriptional regulation of *Il6* required histone deacetylation; inhibition thereof abolished the effect of Tet2 on *Il6* expression, which is in line with previous studies on the role of Tet2 in dendritic cells and macrophages [16,17].

While the role of Tet2 has been studied extensively in the setting of sterile inflammation, chronic diseases and cancer [55], its function in host antibacterial defense is largely unknown. Recent studies investigated the role of Tet2 in the response to LPS in vivo and during abdominal sepsis caused by cecal ligation and puncture making use of mice with an overall genetic deficiency of Tet2 [16,20]. Of note, however, global *Tet2*-knockout mice show dysregulated hematopoietic stem cells, dysfunctional erythroid progenitors and development of myeloid malignancies, as well as abnormal development of immune cells [56,57,58]. In addition, global *Tet2*-knockout mice display features of systemic inflammation as reflected by elevated plasma levels of proinflammatory cytokines such as TNF and IL-12 (14), and had an increased intestinal permeability and spontaneous bacterial translocation from the gut [19]. These constitutive phenotypic deviations associated with global Tet2 deficiency hamper the use of these mice in studies on the role of myeloid cell Tet2 in innate immunity. Therefore, we generated mice with a myeloid cell-specific Tet2 deficiency, by crossing mice in which Tet2 is flanked by two lox-P sites sensitive to excision by Cre-recombinase with mice expressing Cre-recombinase under the myeloid cell-specific LysM promoter [22,59]. The resulting *Tet2^fl/fl^LysM^Cre^* mice are phenotypically normal and show unaltered immune cell development [16]. In global *Tet2*-knockout mice, a systemic LPS challenge resulted in higher IL-6 serum levels when compared with wild-type mice [16]. We here report elevated IL-6 and TNF levels in BALF and PLF of myeloid cell-specific Tet2 deficient mice administered with LPS intranasally or intraperitoneally, respectively, whilst IL-1β and CXCL1 levels did not differ between mouse strains. These findings on the role of Tet2 in LPS-induced cytokine production in different organ compartments in vivo did not fully align with the findings using AMs and PMs in vitro, wherein Tet2 deficiency was associated with increased IL-1β, IL-6 and CXCL1 production without affecting TNF induction. The most likely explanation for the discrepancy between the in vivo and in vitro data is the contribution of different cell types besides macrophages to the production of these mediators in intact animals. Likewise, whilst *E. coli* induced more IL-1β, IL-6 and CXCL1 (but not TNF) in Tet2 deficient PMs than in control PMs, the concentrations of these mediators in PLF only tended to be elevated in *Tet2^fl/fl^LysM^Cre^* mice during *E. coli* peritonitis in vivo. Hence, although Tet2 inhibits the production of various cytokines in tissue macrophages from different organs, its role in mice in vivo is more limited, likely due to cytokine production by other cell types, which may also explain the lack of differences between *Tet2^fl/fl^LysM^Cre^* and control mice in other inflammatory and injurious responses during LPS-induced lung and peritoneal inflammation and *E. coli* peritonitis.

An unexpected finding in the *E. coli* peritonitis model was the increased bacterial growth and dissemination in *Tet2^fl/fl^LysM^Cre^* mice as compared with control mice, which was corroborated by enhanced growth of *E. coli* in cocultures with Tet2 deficient PMs relative to control PMs. In an earlier study, global *Tet2*-knockout and control mice showed similar bacterial loads during polymicrobial sepsis induced by cecal ligation and puncture [20]. As global *Tet2* knockout increases, the myeloid cells that play the most important roles in antibacterial defense in mice, indicating that the antibacterial capacity per cell in *Tet2* knockout host is decreased. Somatic TET2 mutations are among the most frequent reported mutations in human clonal hematopoiesis, which recently has been reported as an increased risk of certain infections caused by both virus and bacteria [60,61,62]. At present, the mechanism by which Tet2 mediates this effect is unknown and is an interesting subject for future investigations.

In summary, we here used myeloid cell-specific Tet2 deficient mice to show that Tet2 acts as a repressor of several proinflammatory cytokines and chemokines in macrophages exposed to LPS or *E. coli*, functioning downstream of MyD88-NFĸB signaling. The impact of myeloid cell Tet2 deficiency during LPS-induced lung and peritoneal inflammation and *E. coli* peritonitis is more limited, most likely through compensation by non-myeloid cells. We recently reported on the role of Tet2 in bronchial epithelial cells in maintaining barrier integrity during pneumonia caused by *Pseudomonas* [26]. These data taken together with recent studies on the role of Tet2 in abdominal sepsis [20] identify a function of Tet2 in acute bacterial infection that stretches beyond myelopoiesis and dysregulations thereof.

## Data Availability

Data are contained within the article or supplementary material.

## Supplementary Materials

The following are available online at https://www.mdpi.com/article/10.3390/cells11010082/s1, The Supplemental Figure S1: Flow cytometry gating strategy to determine the percentage of cell subsets in bronchoalveolar and peritoneal lavage fluid; Figure S2: Tet2 deficiency does not affect 5hmC or the expression of Tet1 and Tet3. Figure S3: Overexpression of TET2 in HEK293T cells. Figure S4: Treatment of TSA or MS-275 does not affect the viability of BMDMs. Figure S5: Myeloid cell specific Tet2 deficiency does not influence lung pathology during LPS induced lung inflammation. Figure S6: The effect of Tet2 deficiency on the expression of antibacterial defense related genes in peritoneal macrophages. Figure S7: The effect of Tet2 deficiency on distant organ damage during *E. coli* induced peritonitis. Table S1: Primers used for RT-qPCR in this study.

## References

[1] Zhang Q., Cao X. (2019). Epigenetic regulation of the innate immune response to infection. Nat. Rev. Immunol..

[2] Fleischmann C., Scherag A., Adhikari N.K., Hartog C.S., Tsaganos T., Schlattmann P., Angus D.C., Reinhart K. (2016). International Forum of Acute Care Trialists. Assessment of Global Incidence and Mortality of Hospital-treated Sepsis. Current Estimates and Limitations. Am. J. Respir. Crit. Care Med..

[3] Cohen J., Vincent J.L., Adhikari N.K., Machado F.R., Angus D.C., Calandra T., Jaton K., Giulieri S., Delaloye J., Opal S. (2015). Sepsis: A roadmap for future research. Lancet Infect. Dis..

[4] Singer M., Deutschman C.S., Seymour C.W., Shankar-Hari M., Annane D., Bauer M., Bellomo R., Bernard G.R., Chiche J.D., Coopersmith C.M. (2016). The Third International Consensus Definitions for Sepsis and Septic Shock (Sepsis-3). JAMA.

[5] Carpenter S., Ricci E.P., Mercier B.C., Moore M.J., Fitzgerald K.A. (2014). Post-transcriptional regulation of gene expression in innate immunity. Nat. Rev. Immunol..

[6] Medzhitov R., Horng T. (2009). Transcriptional control of the inflammatory response. Nat. Rev. Immunol..

[7] Hassan F.I., Didari T., Khan F., Mojtahedzadeh M., Abdollahi M. (2018). The Role of Epigenetic Alterations Involved in Sepsis: An Overview. Curr. Pharm. Des..

[8] Binnie A., Walsh C.J., Hu P., Dwivedi D.J., Fox-Robichaud A., Liaw P.C., Tsang J., Batt J., Carrasqueiro G., Gupta S. (2019). Epigenetic Profiling in Severe Sepsis: A Pilot Study of DNA Methylation Profiles in Critical Illness. Crit. Care Med..

[9] Lorente-Sorolla C., Garcia-Gomez A., Català-Moll F., Toledano V., Ciudad L., Avendaño-Ortiz J., Maroun-Eid C., Martín-Quirós A., Martínez-Gallo M., Ruiz-Sanmartín A. (2019). Inflammatory cytokines and organ dysfunction associate with the aberrant DNA methylome of monocytes in sepsis. Genome Med..

[10] Hopp L., Loeffler-Wirth H., Nersisyan L., Arakelyan A., Binder H. (2018). Footprints of Sepsis Framed Within Community Acquired Pneumonia in the Blood Transcriptome. Front. Immunol..

[11] Pastor W.A., Aravind L., Rao A. (2013). TETonic shift: Biological roles of TET proteins in DNA demethylation and transcription. Nat. Rev. Mol. Cell Biol..

[12] Ichiyama K., Chen T., Wang X., Yan X., Kim B.S., Tanaka S., Ndiaye-Lobry D., Deng Y., Zou Y., Zheng P. (2015). The methylcytosine dioxygenase Tet2 promotes DNA demethylation and activation of cytokine gene expression in T cells. Immunity.

[13] Ma S., Wan X., Deng Z., Shi L., Hao C., Zhou Z., Zhou C., Fang Y., Liu J., Yang J. (2017). Epigenetic regulator CXXC5 recruits DNA demethylase Tet2 to regulate TLR7/9-elicited IFN response in pDCs. J. Exp. Med..

[14] Cull A.H., Snetsinger B., Buckstein R., Wells R.A., Rauh M.J. (2017). Tet2 restrains inflammatory gene expression in macrophages. Exp. Hematol..

[15] Pan W., Zhu S., Qu K., Meeth K., Cheng J., He K., Ma H., Liao Y., Wen X., Roden C. (2017). The DNA Methylcytosine Dioxygenase Tet2 Sustains Immunosuppressive Function of Tumor-Infiltrating Myeloid Cells to Promote Melanoma Progression. Immunity.

[16] Zhang Q., Zhao K., Shen Q., Han Y., Gu Y., Li X., Zhao D., Liu Y., Wang C., Zhang X. (2015). Tet2 is required to resolve inflammation by recruiting Hdac2 to specifically repress IL-6. Nature.

[17] Fuster J.J., MacLauchlan S., Zuriaga M.A., Polackal M.N., Ostriker A.C., Chakraborty R., Wu C.L., Sano S., Muralidharan S., Rius C. (2017). Clonal hematopoiesis associated with TET2 deficiency accelerates atherosclerosis development in mice. Science.

[18] Carrillo-Jimenez A., Deniz Ö., Niklison-Chirou M.V., Ruiz R., Bezerra-Salomão K., Stratoulias V., Amouroux R., Yip P.K., Vilalta A., Cheray M. (2019). TET2 Regulates the Neuroinflammatory Response in Microglia. Cell Rep..

[19] Meisel M., Hinterleitner R., Pacis A., Chen L., Earley Z.M., Mayassi T., Pierre J.F., Ernest J.D., Galipeau H.J., Thuille N. (2018). Microbial signals drive pre-leukaemic myeloproliferation in a Tet2-deficient host. Nature.

[20] Shen Q., Zhang Q., Shi Y., Shi Q., Jiang Y., Gu Y., Li Z., Li X., Zhao K., Wang C. (2018). Tet2 promotes pathogen infection-induced myelopoiesis through mRNA oxidation. Nature.

[21] Quivoron C., Couronné L., Della Valle V., Lopez C.K., Plo I., Wagner-Ballon O., Do Cruzeiro M., Delhommeau F., Arnulf B., Stern M.H. (2011). TET2 inactivation results in pleiotropic hematopoietic abnormalities in mouse and is a recurrent event during human lymphomagenesis. Cancer Cell.

[22] Clausen B.E., Burkhardt C., Reith W., Renkawitz R., Förster I. (1999). Conditional gene targeting in macrophages and granulocytes using LysMcre mice. Transgenic Res..

[23] Abram C.L., Roberge G.L., Hu Y., Lowell C.A. (2014). Comparative analysis of the efficiency and specificity of myeloid-Cre deleting strains using ROSA-EYFP reporter mice. J. Immunol. Methods.

[24] McCubbrey A.L., Allison K.C., Lee-Sherick A.B., Jakubzick C.V., Janssen W.J. (2017). Promoter Specificity and Efficacy in Conditional and Inducible Transgenic Targeting of Lung Macrophages. Front. Immunol..

[25] Weischenfeldt J., Porse B. (2008). Bone Marrow-Derived Macrophages (BMM): Isolation and Applications. CSH Protoc..

[26] Qin W., Brands X., van ‘t Veer C., de Vos A.F., Scicluna B.P., van der Poll T. (2020). Bronchial Epithelial Tet2 Maintains Epithelial Integrity during Acute Pseudomonas aeruginosa Pneumonia. Infect. Immun..

[27] Otto N.A., Butler J.M., Ramirez-Moral I., van Weeghel M., van Heijst J., Scicluna B.P., Houtkooper R.H., de Vos A.F., van der Poll T. (2021). Adherence Affects Monocyte Innate Immune Function and Metabolic Reprogramming after Lipopolysaccharide Stimulation In Vitro. J. Immunol..

[28] Reynier F., de Vos A.F., Hoogerwerf J.J., Bresser P., van der Zee J.S., Paye M., Pachot A., Mougin B., van der Poll T. (2012). Gene expression profiles in alveolar macrophages induced by lipopolysaccharide in humans. Mol. Med..

[29] Namba-Fukuyo H., Funata S., Matsusaka K., Fukuyo M., Rahmutulla B., Mano Y., Fukayama M., Aburatani H., Kaneda A. (2016). TET2 functions as a resistance factor against DNA methylation acquisition during Epstein-Barr virus infection. Oncotarget.

[30] Qin W., Wang L., Zhai R., Ma Q., Liu J., Bao C., Sun D., Zhang H., Sun C., Feng X. (2017). Apa2H1, the first head domain of Apa2 trimeric autotransporter adhesin, activates mouse bone marrow-derived dendritic cells and immunization with Apa2H1 protects against Actinobacillus pleuropneumoniae infection. Mol. Immunol..

[31] Jia Z., Liang Y., Ma B., Xu X., Xiong J., Duan L., Wang D. (2017). A 5-mC Dot Blot Assay Quantifying the DNA Methylation Level of Chondrocyte Dedifferentiation In Vitro. J. Vis. Exp..

[32] Otto N.A., de Vos A.F., van Heijst J., Roelofs J., van der Poll T. (2020). Myeloid Liver Kinase B1 depletion is associated with a reduction in alveolar macrophage numbers and an impaired host defense during gram-negative pneumonia. J. Infect. Dis..

[33] De Porto A.P., Liu Z., de Beer R., Florquin S., de Boer O.J., Hendriks R.W., van der Poll T., de Vos A.F. (2019). Btk inhibitor ibrutinib reduces inflammatory myeloid cell responses in the lung during murine pneumococcal pneumonia. Mol. Med..

[34] Van’t Veer C., van den Pangaart P.S., Kruijswijk D., Florquin S., de Vos A.F., van der Poll T. (2011). Delineation of the role of Toll-like receptor signaling during peritonitis by a gradually growing pathogenic *Escherichia coli*. J. Biol. Chem..

[35] García-Laorden M.I., Stroo I., Terpstra S., Florquin S., Medema J.P., van’t Veer C., de Vos A.F., van der Poll T. (2017). Expression and Function of Granzymes A and B in *Escherichia coli* Peritonitis and Sepsis. Mediat. Inflamm..

[36] Ding C., Scicluna B.P., Stroo I., Yang J., Roelofs J.J., de Boer O.J., de Vos A.F., Nürnberg P., Revenko A.S., Crosby J. (2020). Prekallikrein inhibits innate immune signaling in the lung and impairs host defense during pneumosepsis in mice. J. Pathol..

[37] Tsai W.H., Huang D.Y., Yu Y.H., Chen C.Y., Lin W.W. (2011). Dual roles of NOD2 in TLR4-mediated signal transduction and -induced inflammatory gene expression in macrophages. Cell. Microbiol..

[38] Yang S., Tamai R., Akashi S., Takeuchi O., Akira S., Sugawara S., Takada H. (2001). Synergistic effect of muramyldipeptide with lipopolysaccharide or lipoteichoic acid to induce inflammatory cytokines in human monocytic cells in culture. Infect. Immun..

[39] Wolfert M.A., Murray T.F., Boons G.J., Moore J.N. (2002). The origin of the synergistic effect of muramyl dipeptide with endotoxin and peptidoglycan. J. Biol. Chem..

[40] Kawai T., Akira S. (2011). Toll-like receptors and their crosstalk with other innate receptors in infection and immunity. Immunity.

[41] Wu H., Zhang Y. (2014). Reversing DNA methylation: Mechanisms, genomics, and biological functions. Cell.

[42] Lv L., Wang Q., Xu Y., Tsao L.C., Nakagawa T., Guo H., Su L., Xiong Y. (2018). Vpr Targets TET2 for Degradation by CRL4VprBP E3 Ligase to Sustain IL-6 Expression and Enhance HIV-1 Replication. Mol. Cell.

[43] Mantovani A., Dinarello C.A., Molgora M., Garlanda C. (2019). Interleukin-1 and Related Cytokines in the Regulation of Inflammation and Immunity. Immunity.

[44] Allard B., Panariti A., Martin J.G. (2018). Alveolar Macrophages in the Resolution of Inflammation, Tissue Repair, and Tolerance to Infection. Front. Immunol..

[45] Yona S., Kim K.W., Wolf Y., Mildner A., Varol D., Breker M., Strauss-Ayali D., Viukov S., Guilliams M., Misharin A. (2013). Fate mapping reveals origins and dynamics of monocytes and tissue macrophages under homeostasis. Immunity.

[46] Angus D.C., van der Poll T. (2013). Severe sepsis and septic shock. N. Engl. J. Med..

[47] Mureșan M.G., Balmoș I.A., Badea I., Santini A. (2018). Abdominal Sepsis: An Update. J. Crit. Care Med..

[48] Brook I. (2008). Microbiology and management of abdominal infections. Dig. Dis. Sci..

[49] Cross A., Asher L., Seguin M., Yuan L., Kelly N., Hammack C., Sadoff J., Gemski P. (1995). The importance of a lipopolysaccharide-initiated, cytokine-mediated host defense mechanism in mice against extraintestinally invasive *Escherichia coli*. J. Clin. Investig..

[50] Tsiouplis N.J., Bailey D.W., Chiou L.F., Wissink F.J., Tsagaratou A. (2020). TET-Mediated Epigenetic Regulation in Immune Cell Development and Disease. Front. Cell. Dev. Biol..

[51] Cong B., Zhang Q., Cao X. (2020). The function and regulation of TET2 in innate immunity and inflammation. Protein Cell.

[52] Duployez N., Goursaud L., Fenwarth L., Bories C., Marceau-Renaut A., Boyer T., Fournier E., Nibourel O., Roche-Lestienne C., Huet G. (2020). Familial myeloid malignancies with germline TET2 mutation. Leukemia.

[53] Kaasinen E., Kuismin O., Rajamäki K., Ristolainen H., Aavikko M., Kondelin J., Saarinen S., Berta D.G., Katainen R., Hirvonen E. (2019). Impact of constitutional TET2 haploinsufficiency on molecular and clinical phenotype in humans. Nat. Commun..

[54] Stetson D.B., Medzhitov R. (2006). Type I interferons in host defense. Immunity.

[55] Jiang S. (2020). Tet2 at the interface between cancer and immunity. Commun. Biol..

[56] Li Z., Cai X., Cai C.L., Wang J., Zhang W., Petersen B.E., Yang F.C., Xu M. (2011). Deletion of Tet2 in mice leads to dysregulated hematopoietic stem cells and subsequent development of myeloid malignancies. Blood.

[57] Ito K., Lee J., Chrysanthou S., Zhao Y., Josephs K., Sato H., Teruya-Feldstein J., Zheng D., Dawlaty M.M., Ito K. (2019). Non-catalytic Roles of Tet2 Are Essential to Regulate Hematopoietic Stem and Progenitor Cell Homeostasis. Cell Rep..

[58] Qu X., Zhang S., Wang S., Wang Y., Li W., Huang Y., Zhao H., Wu X., An C., Guo X. (2018). TET2 deficiency leads to stem cell factor-dependent clonal expansion of dysfunctional erythroid progenitors. Blood.

[59] Van Lieshout M.H., Anas A.A., Florquin S., Hou B., van’t Veer C., de Vos A.F., van der Poll T. (2014). Hematopoietic but not endothelial cell MyD88 contributes to host defense during gram-negative pneumonia derived sepsis. PLoS Pathog..

[60] Bolton K.L., Koh Y., Foote M.B., Im H., Jee J., Sun C.H., Safonov A., Ptashkin R., Moon J.H., Lee J.Y. (2020). Clonal hematopoiesis is associated with risk of severe Covid-19. medRxiv.

[61] Duployez N., Demonchy J., Berthon C., Goutay J., Caplan M., Moreau A.S., Bignon A., Marceau-Renaut A., Garrigue D., Raczkiewicz I. (2020). Clinico-Biological Features and Clonal Hematopoiesis in Patients with Severe COVID-19. Cancers.

[62] Hameister E., Stolz S.M., Fuhrer Y., Thienemann F., Schaer D.J., Nemeth J., Schuepbach R.A., Goede J., Reinhart S., Schmidt A. (2020). Clonal Hematopoiesis in Hospitalized Elderly Patients With COVID-19. HemaSphere.

