# Supplementary material for: Role of Myeloid Tet Methylcytosine Dioxygenase 2 in Pulmonary and Peritoneal Inflammation Induced by Lipopolysaccharide and Peritonitis Induced by Escherichia coli
Source: Cells. 2021 Dec 28;11(1):82. doi: 10.3390/cells11010082 (PMC8750455; doi:10.3390/cells11010082)

cells

MDPI

Supplementary materials

Article

Role of Myeloid Tet Methylcytosine Dioxygenase 2 in Pulmonary and Peritoneal Inflammation Induced by Lipopolysaccharide and Peritonitis Induced by *Escherichia coli*

Wanhai Qin [1,*], Xanthe Brands [1], Hisatake Matsumoto [1], Joe M. Butler [1], Cornelis van't Veer [1], Alex F. de Vos [1], Joris J. T. H. Roelofs [2], Brendon P. Scicluna [1,3] and Tom van der Poll [1,4]

[1] Center of Experimental & Molecular Medicine, Amsterdam University Medical Centers, location Academic Medical Center, University of Amsterdam, 1105AZ Amsterdam, The Netherlands; x.brands@amsterdamumc.nl (X.B.); h.matsumoto0828@gmail.com (H.M.); j.m.butler@amsterdamumc.nl (J.M.B.); c.vantveer@amsterdamumc.nl (C.v.V.); a.f.devos@amsterdamumc.nl (A.F.d.V.); b.scicluna@amsterdamumc.nl (B.P.S.); t.vanderpoll@amsterdamumc.nl (T.v.d.P.)

[2] Department of Pathology, Amsterdam University Medical Centers, location Academic Medical Center, University of Amsterdam, 1105AZ Amsterdam, The Netherlands; j.j.roelofs@amsterdamumc.nl

[3] Department of Clinical Epidemiology, Biostatistics and Bioinformatics, Amsterdam University Medical Centers, location Academic Medical Center, University of Amsterdam, 1105AZ Amsterdam, The Netherlands

[4] Division of Infectious Diseases, Amsterdam University Medical Centers, location Academic Medical Center, University of Amsterdam, Meibergdreef 9, 1105 AZ Amsterdam, The Netherlands

* Correspondence: w.qin@amsterdamumc.nl

Citation: Qin, W.; Brands, X.; Matsumoto, H.; Butler, J.M.; van't Veer, C.; de Vos, A.F.; Roelofs, J.J.T.H.; Scicluna, B.P.; van der Poll, T. Role of Myeloid Tet Methylcytosine Dioxygenase 2 in Pulmonary and Peritoneal Inflammation Induced by Lipopolysaccharide and Peritonitis Induced by *Escherichia coli*. *Cells* **2022**, *11*, 82. https://doi.org/10.3390/cells11010082

Academic Editor: Alessandro Poggi

Received: 23 August 2021
Accepted: 24 December 2021
Published: 28 December 2021

Publisher's Note: MDPI stays neutral with regard to jurisdictional claims in published maps and institu-tional affiliations.

Copyright: © 2021 by the authors. Licensee MDPI, Basel, Switzerland. This article is an open access article distributed under the terms and conditions of the Creative Commons Attribution (CC BY) license (https://creativecommons.org/licenses/by/4.0/).

Supplementary table

Table S1. Primers used for RT-qPCR in this study.

Gene	Forward	Reverse
Mouse *Cxcl1*	CCACTGCACCCAAACCGAAG	TCCGTTACTTGGGGACACCT
Mouse *Il1b*	GGGGAACTCTGCAGACTCAA	GGGCCTCAAAGGAAAGAATC
Mouse *Il6*	CTTCCTACCCCAATTTCCAATGCT	TCTTGGTCCTTAGCCACTCCTT
Mouse *Tnf*	CGAGTGACAAGCCTGTAGCC	CCTTGAAGAGAACCTGGGAGT
Mouse *Ifnb*	GCACTGGGTGGAATGAGACTATTG	TTCTGAGGCATCAACTGACAGGTC
Mouse *Tet2*	AGCTGATGGAAAATGCAAGC	AAGGTGCCTCTGGAGTGTTG
Mouse *Tet1*	GAGCCTGTTCCTCGATGTGG	CAAACCCACCTGAGGCTGTT
Mouse *Tet3*	TCCGGATTGAGAAGGTCATC	CCAGGCCAGGATCAAGATAA
Mouse *Nos2*	GTGGACGGGTCGATGTCAC	GTTCTCAGCCCAACAATAC
Mouse *Rnase6*	CTGTGGGAGCCGATGTATCTA	TTGCATGGTTGACGACTTGTC
Mouse *Lcn2*	GCAGGTGGTACGTTGTGGG	CTCTTGTAGCTCATAGATGGTGC
Mouse *Hamp*	TGCAGAAGAGAAGGAAGA-GAGACA	CACACTGGGAATTGTTACAGCATT
Mouse *Camp*	GCTGTGGCGGTCACTATCAC	TGTCTAGGGACTGCTGGTTGA
Mouse *Cat*	GCGTCCAGTGCGCTGTAGA	TCAGGGTGGACGTCAGTGAA
Mouse *Nox2*	CCCTTTGGTACAGCCAGTGAAGAT	CAATCCCGGCTCCCACTAACATCA
Mouse *Sod1*	TGGGTTCCACGTCCATCAGTA	ACCGTCCTTTCCAGCAGTCA
Mouse *Ptx3*	CCTGCTTTGTGCTCTCTGGT	TCTCCAGCATGATGAACAGC
Mouse *Gpx4*	TCTGGCAGGCACCATGTGT	CGGGCATGCAGATCGACTA
Mouse *Hprt*	AGTCAAGGGCATATCCAACA	CAAACTTTGCTTTCCGGGT

Figure S1. Flow cytometry gating strategy to determine the percentage of cell subsets in bronchoalveolar and peritoneal lavage fluid. (**A**) Gating strategy to determine the percentage of alveolar macrophages (AMs: CD45+Siglec F+CD11b-), and neutrophils (Neu: CD45+Siglec F+CD11b+Ly6C+Ly6G+) in BALF. (**B**) Gating strategy to determine the percentage of peritoneal macrophages (PMs: CD45+F4/80+CD11b+), monocytes (Mono: CD45+F4/80-CD11b+Ly6C+Ly6G-) and neutrophils (Neu: CD45+F4/80-CD11b+Ly6C+Ly6G+) in PLF.

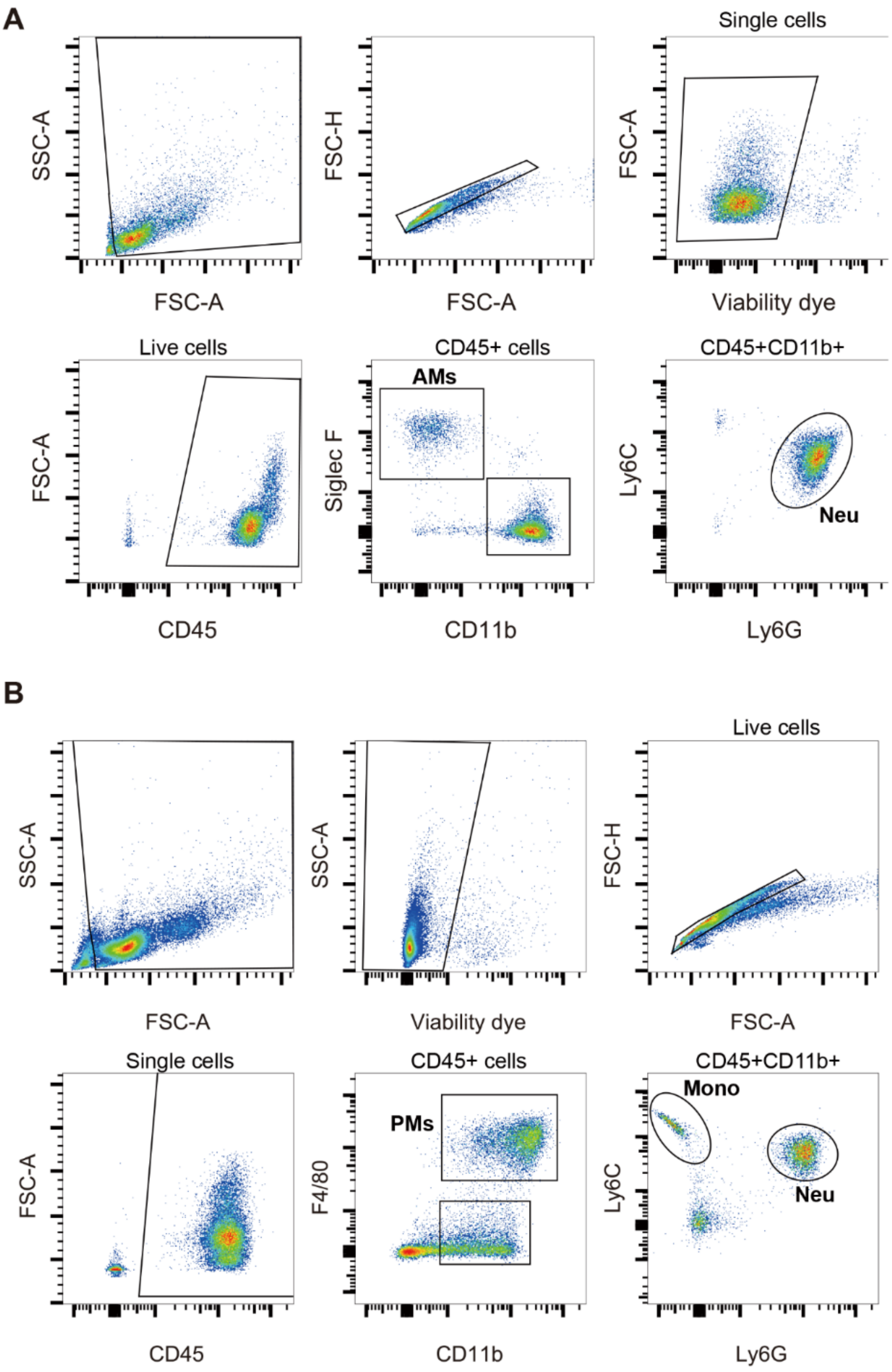

Figure S2. Tet2 deficiency does not affect 5hmC or the expression of *Tet1* and *Tet3*. (**A**) The same amount of loading DNA from BMDMs was validated by methylene blue straining. (**B**) 5-hmC levels of in DNA isolated from naive BMDMs was measured by Global DNA hydroxymethylation (5-hmC) ELISA assay. (**C**) The expression of Tet1 and Tet3 in naive BMDMs was measured by qPCR. n = 3 for **A** and n = 6 for **B**-**C**. Data is shown as bar graphs with mean ± SEM. Differences between groups were not significant.

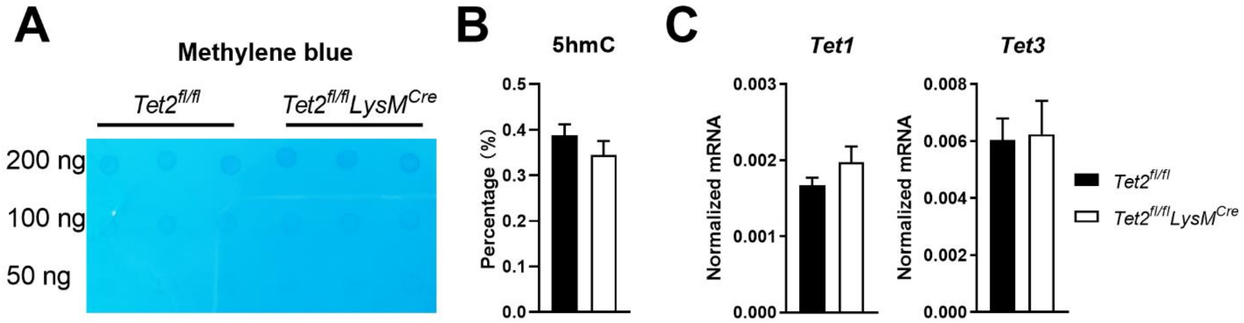

Figure S3. Overexpression of TET2 in HEK293T cells. TET2 levels measured by western blot in HEK293T cells co-transfected MyD88 with increasing amount of TET2 expression vector (0, 30 or 150 ng) 24 hours earlier. CTRL = cells transfected with empty vector, TET2 OE = cells transfected with TET2 plasmid. .

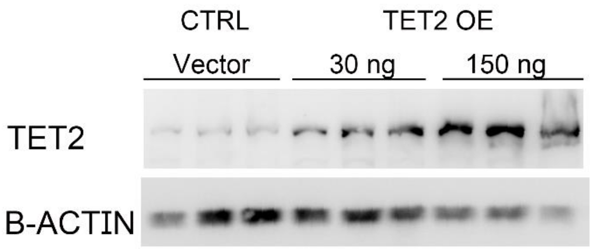

Figure S4. Treatment of TSA or MS-275 does not affect the viability of BMDMs. BMDMs were pre-treated (2 hours) with 100 nM trichostatin A, 4 μM MS-275 or vehcile before stimulation with LPS (100 ng/ml) or medium control for another 12 hours; BMDMs were then collected and stained with fixable viability dye eFluo 780 for measuring cell viability using flow cytometry. $n = 6$. Data is shown as bar graphs with mean ± SEM. Differences between groups were not significant.

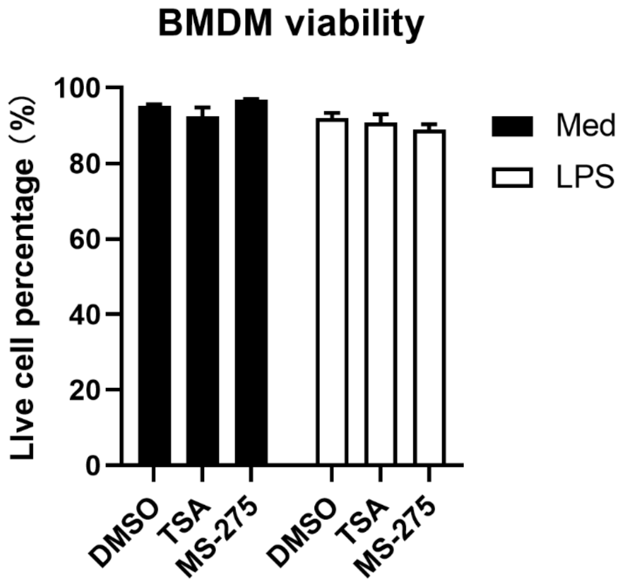

Figure S5. Myeloid cell specific Tet2 deficiency does not influence lung pathology during LPS induced lung inflammation. *Tet2[fl/fl]LysM[Cre]* and control *Tet2[fl/fl]* mice were administered with LPS (1 mg/kg) via the airways and lung tissue was collected 6 hours later. (**A**) Representative photographs of H&E-stained lung sections (magnification: 20x); (**B**) Lung pathology scored according to a semi-quantitative scoring system described in the Methods. Bar graphs show data as means ± SEM of 8 mice per group. Differences between groups were not significant.

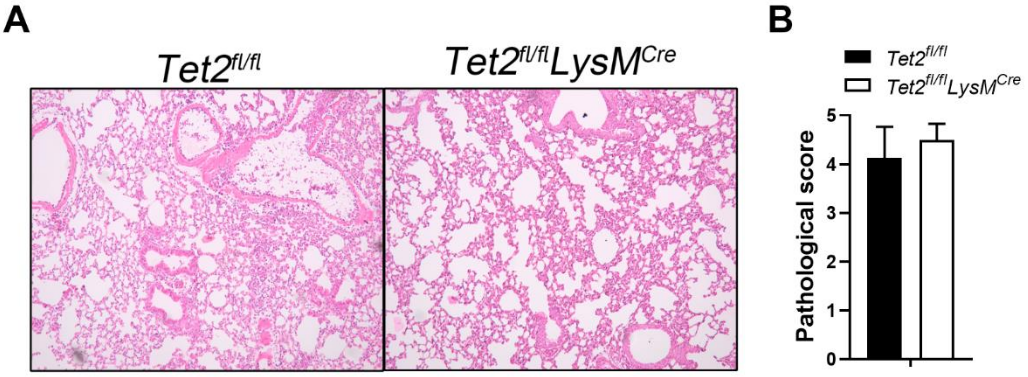

Figure S6. The effect of Tet2 deficiency on the expression of antibacterial defense related genes in peritoneal macrophages. Peritoneal macrophages were stimulated with heat killed *E. coli* for 12 hours. (**A**) The expression of *Rnase6* (encoding ribonuclease A family member K6), *Lcn2* (lipocalin-2), *Hamp* (hepcidin antimicrobial peptide), *Camp* (cathelicidin antimicrobial peptide) and *Cat* (catalase), (**B**) *Nos2* (nitric oxide synthase 2), *Nox2* (NADPH oxidase 2), *Sod1* (superoxide dismutase), *Gpx4* (glutathione peroxidase 4) and *Ptx3* (pentraxin 3) was evaluated by qPCR. Bar graphs show data as means ± SEM. *n* =6. Differences between groups were not significant.

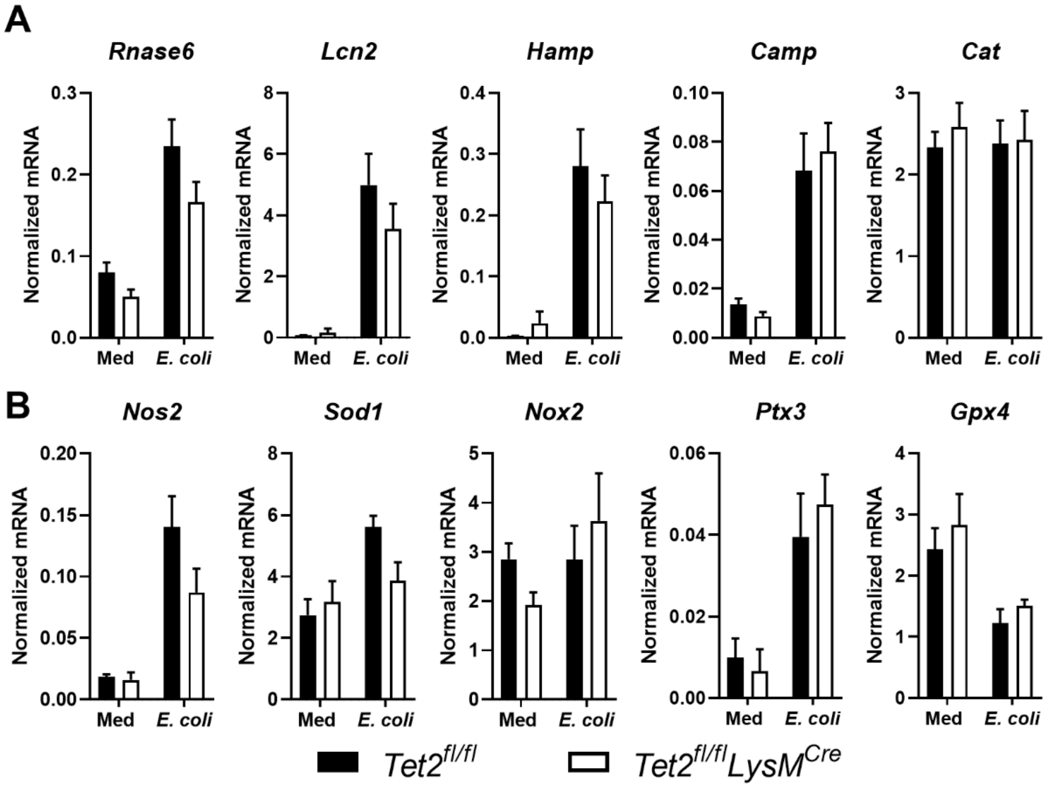

Figure S7. The effect of Tet2 deficiency on distant organ damage during *E. coli* induced peritonitis. $Tet2^{fl/fl}LysM^{Cre}$ (open bars) and control $Tet2^{fl/fl}$ mice (black bars) were infected with *E. coli* (10^4 CFU) intraperitoneally; plasma and lungs were collected 16 hours later. (**A**) AST, (**B**) ALT and (**C**) LDH levels in plasma; (**D**) Representative photographs of H&E-stained lung sections (magnification: 20x); (**E**) Lung pathology scored according to a semi-quantitative scoring system described in the Methods. Bar graphs show data as means ± SEM. *n*=8. Differences between groups were not significant.

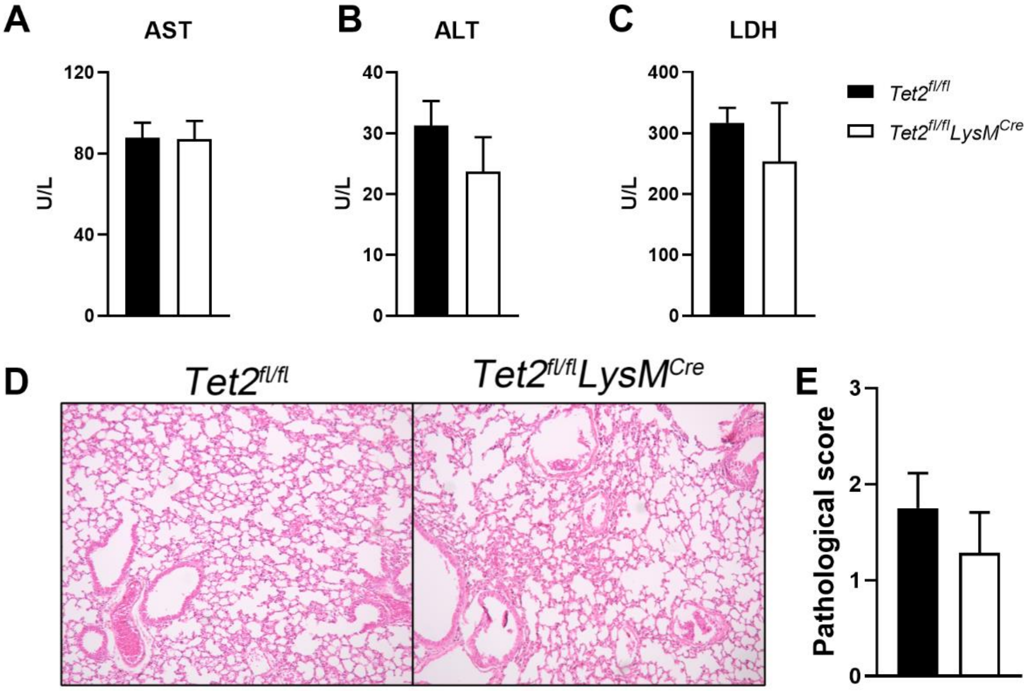

Supplement: Supplementary file 1 [file cells-11-00082-s001.zip › cells-1371333-supplementary.pdf]
